# Statement of Retraction: Asiatic acid re-sensitizes multidrug-resistant A549/DDP cells to cisplatin by down regulating long non-coding RNA metastasis associated lung adenocarcinoma transcript 1/β-catenin signaling

**DOI:** 10.1080/21655979.2026.2731784

**Published:** 2026-10-01

We, the Editors and Publisher of the journal *Bioengineered*, have retracted the following article from publication. 

Cheng, Q., Zhang, S., Zhong, B., Chen, Z., & Peng, F. (2022). Asiatic acid re-sensitizes multidrug-resistant A549/DDP cells to cisplatin by down regulating long non-coding RNA metastasis associated lung adenocarcinoma transcript 1/β-catenin signaling. *Bioengineered*, *13*(5), 12972–12984. https://doi.org/10.1080/21655979.2022.2079302

Since publication, significant concerns have been raised about the integrity of the data and reported results in the article.

When approached for an explanation, the authors have not responded to our queries and so these serious concerns remain unaddressed.

As verifying the validity of published work is core to the integrity of the scholarly record, we are therefore retracting the article. The authors have been informed of this decision.

We have been informed in our decision-making by our policy on publishing ethics and integrity and COPE guidelines.  

The retracted article will remain online to maintain the scholarly record and will be digitally watermarked on each page as ‘Retracted’.  

